# Single wall carbon nanotubes enter cells by endocytosis and not membrane penetration

**DOI:** 10.1186/1477-3155-9-45

**Published:** 2011-09-30

**Authors:** Peter N Yaron, Brian D Holt, Philip A Short, Mathias Lösche, Mohammad F Islam, Kris Noel Dahl

**Affiliations:** 1 Department of Chemical Engineering, Carnegie Mellon University, Pittsburgh, PA, USA; 2 Department of Biomedical Engineering, Carnegie Mellon University, Pittsburgh, PA, USA; 3 Department of Physics, Carnegie Mellon University, Pittsburgh, PA, USA; 4 Center for Neutron Research, The National Institute for Standards and Technology, Gaithersburg, MD, USA; 5 Department of Materials Science & Engineering, Carnegie Mellon University, Pittsburgh, PA, USA

## Abstract

**Background:**

Carbon nanotubes are increasingly being tested for use in cellular applications. Determining the mode of entry is essential to control and regulate specific interactions with cells, to understand toxicological effects of nanotubes, and to develop nanotube-based cellular technologies. We investigated cellular uptake of Pluronic copolymer-stabilized, purified ~145 nm long single wall carbon nanotubes (SWCNTs) through a series of complementary cellular, cell-mimetic, and in vitro model membrane experiments.

**Results:**

SWCNTs localized within fluorescently labeled endosomes, and confocal Raman spectroscopy showed a dramatic reduction in SWCNT uptake into cells at 4°C compared with 37°C. These data suggest energy-dependent endocytosis, as shown previously. We also examined the possibility for non-specific physical penetration of SWCNTs through the plasma membrane. Electrochemical impedance spectroscopy and Langmuir monolayer film balance measurements showed that Pluronic-stabilized SWCNTs associated with membranes but did not possess sufficient insertion energy to penetrate through the membrane. SWCNTs associated with vesicles made from plasma membranes but did not rupture the vesicles.

**Conclusions:**

These measurements, combined, demonstrate that Pluronic-stabilized SWCNTs only enter cells via energy-dependent endocytosis, and association of SWCNTs to membrane likely increases uptake.

## Background

Carbon nanotubes (CNTs) have recently been explored for potential uses in biology and medicine. Their small size, high surface area, inert chemical composition, and unique physical properties have made them extensively investigated for transport of DNA[1], nucleic acids[2], drugs[3], and a variety of other potential therapeutics[4]. Single wall CNTs (SWCNTs) with a 1-2 nm outer diameter have variable length and unique optical and electrical properties[5] desirable for biological applications[6]. Cytotoxicity of SWCNTs depends on SWCNT length, impurities, and dispersion quality (isolated vs. bundles)[7]. SWCNTs dispersed in a biocompatible Pluronic triblock copolymer reorganize sub-cellular structures without inducing cell death[8,9]. To better understand the toxicological effects posed by SWCNTs and to develop SWCNT-related cellular biotechnologies, unambiguous determination of the mechanism of uptake into the cell is essential.

Competing hypotheses exist regarding the mechanism by which SWCNTs enter cells: non-specific physical penetration of the cell membrane, endocytosis or both. Numerous studies have imaged CNTs inside cells and have shown that CNTs are endocytosed[10-14]. Theoretical and simulation studies on CNT uptake into cells provided contradictory results: some theoretical reports have suggested that CNTs may not be able to trigger endocytosis due to their small diameter and the kinetics of endosome formation[15,16]. Simulation studies have shown that CNTs have affinity for membranes[17], but suggest that CNTs have insufficient energy to pierce through both leaflets of a membrane. While endocytosis is the commonly suggested mechanism of cellular uptake, physical penetration has not been rigorously considered and may account for significant uptake. In particular, alteration or disruption of sub-cellular membranous structures or CNT affinity to membranes may also be responsible for altering cellular uptake and architecture.

Here, we employed complementary methods including in vitro model membranes and cellular imaging to investigate mechanisms of cellular uptake of short (145 ± 17 nm), Pluronic F-127 (PF-127) triblock copolymer-dispersed SWCNTs in Millipore-filtered deionized water to discern whether cellular uptake occurs only via an active endocytosis process or passive physical penetration through the membrane. To minimize confounding effects due to SWCNT sample preparation, such as contamination from metal catalysts, undesirable carbon polymorphs, distribution of SWCNT lengths, and defects resulting from functionalization methods, we utilized highly purified, length-selected, dispersed, pristine SWCNTs that have been previously developed in our group[9,18,19]. We have determined uptake and localization of Pluronic copolymer-stabilized SWCNTs into cells through temperature dependent cell studies with confocal Raman spectroscopy and fluorescence lifetime imaging (FLIM). We also used electrochemical impedance spectroscopy (EIS) of sparsely-tethered bilayer lipid membranes (stBLMs) and Langmuir monolayers (LMs) of synthetic phospholipids to model the plasma membrane. We further verified our results by examining interactions between SWCNTs and giant plasma membrane vesicles (GPMVs). GPMVs are produced from cell membranes, are more complex than synthetic lipid systems, but circumvent biological complications of cells. stBLMs, LMs and GPMVs have been utilized previously as model systems with great success to determine spatial localization, binding affinities, dissociation constants, and insertion pressures necessary for membrane association and cellular incorporation of materials[20-22].

## Results

### SWCNTs added to the extracellular media localize within cells

**Figure 1:**
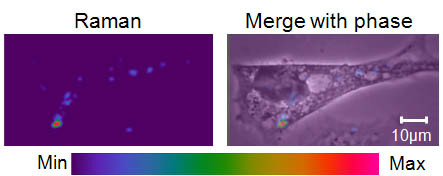
**SWCNTs localized within cells visualized by confocal Raman spectroscopy**. A confocal Raman image (with 100 × oil objective, data acquired in 1 μm increments) was acquired of the focal plane of a fixed, hematoxylin-labeled HeLa cell that had been exposed to SWCNTs for 2 days. The left image shows the spatial distribution of the SWCNT (G-band) Raman intensity, and the right image superimposes the Raman intensity onto a phase contrast image. SWCNT intensity was sub-cellularly localized, preferentially in the perinuclear regions and the cytoplasm. This image is representative of more than 10 imaged cells.

We confocally imaged the Raman spectra inside HeLa cells to visualize the sub-cellular localization of PF-127 stabilized SWCNTs. The Raman intensity distribution, obtained from the G-band (1590 cm^-1^) [13,23], shows SWCNT concentration localized to the mid-plane of a fixed, hematoxylin-labeled cell (Figure 1). SWCNTs were preferentially located within cells versus the extracellular regions, and most SWCNTs were localized at the perinuclear region (Figure 1). Similar cellular localization of SWCNTs has been previously observed[10,13,24].

### Lipid bilayer association of SWCNTs

To determine if SWCNTs perforate bilayers, and by a similar mechanism might penetrate the plasma membrane of the cell, we examined SWCNT interactions with synthetic lipid bilayers. We monitored the electric responses of sparsely-tethered bilayer lipid membranes (stBLMs) to SWCNT addition using electrochemical impedance spectroscopy (EIS; Additional Methods and Additional Figure 1A). It was recently shown that stBLMs prepared by rapid solvent exchange[25] form complete bilayers on solid substrates that are contiguous and virtually defect-free[25]. These stBLMs are decoupled from the substrates by a highly hydrated, nanometer-thin stratum to retain their in-plane fluidity and, therefore, constitute realistic models of lipid membranes[22]. Specifically, they form fluid, disordered membranes with a resistance in the MΩcm^2 ^regime[24] that permits the sensitive detection of changes in their electrical properties by membrane-targeting enzymes[26] or channel-forming toxins[27,28].

**Figure 2:**
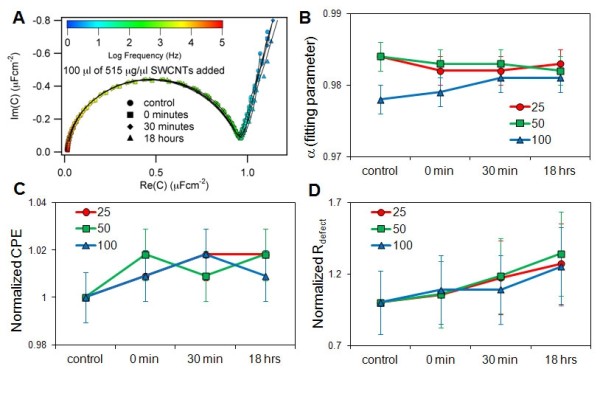
**SWCNTs and PF-127 do not alter membrane capacitance of stBLM**. **(A) **EIS data was analyzed as a Cole-Cole plot of the stBLM before and after exposure to SWCNTs dispersion. The imaginary versus the real (Im(*C*) vs. Re(*C*)) parts of capacitance sweeps from 0.1 Hz to 60,000 Hz were plotted as an overlaid color spectrum. Exposure to SWCNTs showed little change to the stBLM indicating no penetration or intercalation into the membrane leaflets. **(B) **Model fit of the ECM showed little change in α (α > 0.95), suggesting a near perfect membrane. **(C **and **D) **Model fit of the normalized CPE_stBLM _and *R*_defect _values showed little change in membrane properties after treatment. As a comparison, disruption or perforation of membranes changes R_defect _by more than 100 ×.

SWCNTs dispersed with PF-127 were added to stBLMs prepared from DOPC to determine changes in membrane capacitance and resistance. EI spectra of the stBLM were collected at 0 minutes, 20 minutes and 18 hours for different concentrations of PF-127-stabilized SWCNTs and PF-127 without SWCNTs. The spectra are displayed as Cole-Cole plots (Figure 2A), which exhibited a semicircular shape, consistent with the capacitive properties of a near-ideally insulating dielectric layer (Figure 2A). Spectra were fit (solid lines) to an equivalent circuit model (ECM) that is effective for modeling dielectric properties of highly insulating solid-supported phospholipid bilayers[29] (see Methods, Additional Methods, and Additional Figure 1B). To compare the effects of SWCNTs on the bilayers, we normalized the parameters obtained from the fits to those obtained for the stBLM prior to SWCNT addition.

The results (Figure 2B-D and Additional Information) showed that the stBLMs possess near-ideal capacitive behavior. The exponents of the constant phase element (CPE; see Methods and Additional Methods) were always near unity with α_stBLM _> 0.95, (Figure 2B) allowing the stBLM CPE in the ECM to be approximated as a capacitance CPE_stBLM _≈ *C*_stBLM_. Tracking *C*_stBLM _versus incubation time (Figure 2C) or concentration (Additional Information) showed a slight increase, indicating a small change in local dielectric constant in the hydrophobic core or in bilayer thickness. The second CPE in the ECM (see Additional Figure 1B), CPE_defect_, depends on electrical conductivity of the sub-membrane space. Increased electric resistance of the aqueous reservoir leads to a higher electric field penetration into this space and, consequently, a smaller CPE_defect_. Assuming that ion mobility remains the same in the sub-membrane space as in the bulk of the electrolyte, this defect density in the stBLM may be estimated from *R*_defect_. Changes in *R*_defect _versus incubation time (Figure 2D) and concentration (Additional Information) were negligible, suggesting no change in bilayer defect density due to changes induced by incubation with SWCNTs. For comparison, R_defect _may change by more than two orders of magnitude when stBLMs are perforated by protein membrane pores or reduced in their thickness, leading to a lower hydrophobic barrier to ion transfer across the bilayer[27,28]. While the SWCNTs did not affect the capacitance or resistance of the membrane significantly, there were long-term changes in the membrane properties in the presence of PF-127 and SWCNTs suggesting some reordering of the membranes.

### Interaction of SWCNTs with Langmuir monolayers

**Figure 3:**
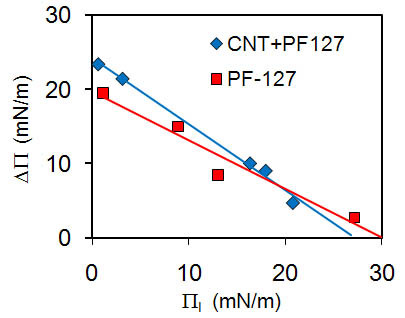
**SWCNTs associate with membranes but not with sufficient energy to penetrate the layer**. The initial monolayer pressure, Π_i_, was altered (increased by ΔΠ) by the addition of SWCNTs or PF-127. The MIP is defined as ΔΠ = 0 (ΔΠ crosses the x-axis). MIP for SWCNTs and PF-127 (Table 1) were close to but less than the insertion pressure of 30 - 35 mN/m required to penetrate a bilayer[31].

To test if SWCNTs partially incorporate into the membrane[17] we investigated the interaction of SWCNTs with Langmuir monolayers (LMs). LMs, although only one half of a lipid bilayer, allow quantification of the thermodynamics of the membrane interface with an aqueous environment[30-32]. Specifically, LMs allow control of the surface pressure, Π and the interfacial energy, γ, via adjustments of the interfacial area, A, within the Langmuir trough. At low surface pressures, which correspond to high surface energy (Π = γ_0 _- γ, where γ_0 _is the energy of the bare aqueous surface), penetration of a material into the lipid film is strongly favored. Incubation of a pre-formed phospholipid LM at constant area A_0 _therefore leads to an increase of the initial surface pressure from Π_i _= Π (A_0_) to Π_i _+ ΔΠ due to incorporation of the adsorbent. The observed value of ΔΠ depends on the interaction of the adsorbent with the membrane interface and is reduced if the initial value of Π is increased in a series of experiments. For protein adsorbents, the reduction in ΔΠ is approximately linear in response to increases in Π(A_0_) and can be extrapolated to ΔΠ = 0 to determine the maximum insertion pressure (MIP), Π*, *i.e*. the initial surface pressure at which no more adsorbent can penetrate the LM[20]. It is commonly assumed that an adsorbent will likely insert into bilayer membranes of the same composition as the LM if Π* > 30-35 mN/m because this is the approximate value at which a LM is in a thermodynamic state equivalent to that of the respective bilayer membrane[31,33,34]. MIP experiments were carried out with SWCNTs stabilized with PF-127 polymer and with the PF-127 polymer alone using DPPC in a LM (Figure 3). Extrapolation of ΔΠ vs. Π_i _yielded MIP values just below the bilayer-equivalent pressure, 28 - 30 mN/m (Table 1). This is consistent with previous reports that Pluronics (poloxamers) are able insert into membranes and alter membrane fluidity and membrane defects[35].

**Table 1:** MIP calculated from linear fits of DPPC monolayers exposed to SWCNTs dispersed in PF-127 and to PF-127 alone.

	MIP (mN/m)	R^2^
**PF-127**	30.0	0.946
**SWCNT**	28.9	0.993

At room temperature, DPPC forms phase-separated LMs in a coexistence regime, which complicates the determination of the SWCNTs' MIP[20]. To confirm MIP results in a purely fluid lipid phase, similar experiments were performed using DOPC LMs. However, due to its higher fluidity than DPPC, DOPC LMs are intrinsically less stable, making reproducibility an issue[32]. We never observed any pressure increase ΔΠ at initial pressures exceeding 22 mN/m, but we were unable to produce a MIP plot of similar accuracy to that obtained using DPPC.

PF-127 has a higher MIP than the PF-127 stabilized SWCNTs (Table 1), suggesting that the free polymer has a higher affinity for the membrane. To determine if the PF-127 dispersant was separating from the SWCNTs and formed aggregates in the experiments, we took samples from the subphase of the Langmuir film balance after experiments and checked for SWCNT aggregates using Vis-NIR absorbance spectroscopy. There was no significant change observed compared with a freshly-dispersed nanotube solution.

### FLIM of CellMask orange-labeled GPMVs with SWCNTs

To determine if SWCNTs have a preferential association with bilayer membranes and to provide a link between synthetic lipid and cell experiments, we visualized SWCNT interactions with vesicles derived from cell plasma membranes. GPMVs, produced from NIH-3T3 cells (see Methods), were labeled with CellMask orange and exposed to SWCNTs at similar time points and concentration as the other experiments. GPMVs were imaged via widefield microscopy for > 2 hours, and no vesicle rupture was observed.

**Figure 4:**
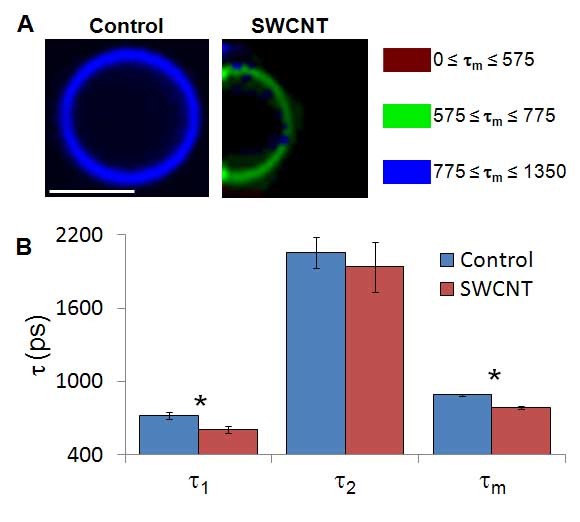
**FLIM of CellMask orange labeled GPMVs exposed to SWCNTs**. **(A) **Addition of SWCNTs to GPMVs synthesized from NIH-3T3 cells was associated with a decrease in average fluorescence lifetime (τ_m_) of CellMask orange. **(B) **Statistical decrease in both τ_1 _and τ_m _represent a SWCNT-based quenching of CellMask fluorescence (* is p < 0.01).

We performed fluorescence lifetime imaging microscopy (FLIM) of the CellMask orange with the SWCNTs. When fluorophores are excited with a femtosecond excitation pulse, the emission photons are detected after excitation and the distribution can be modeled as a single- or multi-exponential decay curve with characteristic time constant(s), τ. FLIM is sensitive to the local (~5 nm) nanoenvironment including proximity to quenching molecules but is independent of fluorophore concentration, photobleaching and excitation light intensity [36]. CellMask orange was best modeled with a double exponential decay, as judged from the observation of improved fits to a double exponential decay without over-parameterization (χ^2 ^closest to unity) and dissimilar time constants with non-statistical amplitudes[36]. SWCNT-treated samples showed a statistically significant reduction in mean fluorescence lifetime, τ_m_, compared to control (Figure 4A, B and Table 2). For τ_1_, τ_2 _and τ_m_, the SWCNT-treated samples possessed a shorter lifetime (p < 0.01 for τ_1 _and τ_m_), suggesting that SWCNTs were sufficiently close to the membrane label to quench the fluorescence lifetime of the CellMask orange.

**Table 2:** Fluorescence lifetime imaging microscopy (FLIM) values obtained from CellMask Orange-treated GPMVs exposed to SWCNTs.

FLIM of GPMVs with CellMask Orange (+) SWCNTs						
	τ_1_	A_1_	τ_2_	A_2_	τ_m_	χ^2^
**Control**	721 ± 90	0.829 ± 0.186	2055 ± 464	0.171 ± 0.066	890 ± 32	1.357 ± 0.089
**30 min**.	505 ± 62	0.859 ± 0.099	1861 ± 462	0.141 ± 0.032	674 ± 20	1.462 ± 0.073

### Temperature-dependent endocytosis assay

**Figure 5:**
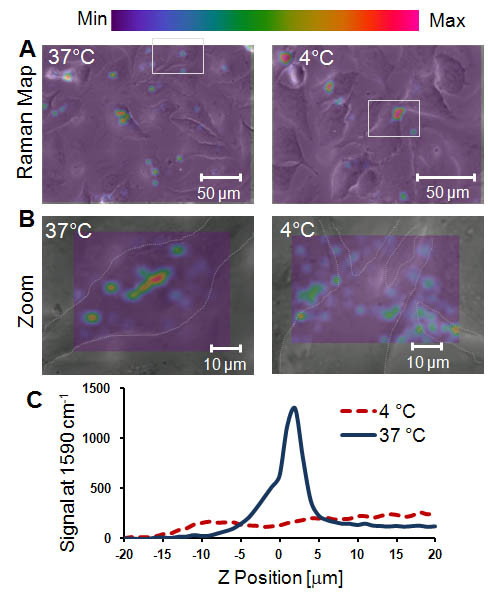
**Temperature-dependent uptake of SWCNTs measured with confocal Raman spectroscopy**. **(A) **Cells were exposed to SWCNTs for 45 min at either 37°C or 4°C to test for endocytosis, which is an active cellular process. At 37°C, SWCNTs localized within cells; and at 4°C, SWCNTs localized outside cells, as measured by Raman (with 40 × oil objective, data acquired in 5 μm increments). **(B) **Zooming into the cell (cell outline sketched with a dashed line), we observed a colocalization of SWCNT Raman signal within cells at 37°C versus excluded from cells at 4°C (with 100 × oil objective, data acquired in 1 μm increments). **(C) **From part C, we integrated the Raman signal over x-y and plotted as a function of z-position within the cell. At 37°C we observed increased Raman signal from -2 to 5 μm, which corresponds to a region within the cell. At 4°C, there was no increase in Raman signal. More than 25 cells were imaged and averaged for each condition.

We have shown with *in vitro *assays that SWCNTs do not penetrate either synthetic or plasma membrane derived bilayers. To perform the analogous experiments in cells, we obtained confocal Raman spectra of HeLa cells that were maintained at either 37°C or 4°C (Figure 5A). At 4°C, active cellular processes including endocytosis are inhibited. These cells were not labeled with hematoxylin so sub-cellular features are not clearly visible in phase-contrast images. SWCNTs interacted with the cells at both 37°C and 4°C after only 45 minutes of incubation, but the SWCNT G-band Raman intensity was stronger for the 37°C, sample (Figure 5A). Zooming into regions of SWCNT intensity (boxes) revealed that cellular localization of SWCNTs was different for the 37°C and 4°C samples (Figure 5B). At 37°C SWCNTs were primarily contained within the cell (dashed to enhance cellular borders). The 4°C sample showed SWCNT intensity preferentially located outside of and at the edge of the cell with only a small fraction interacting with the cell (Figure 5B).

To quantify SWCNT distribution in the cells at 37°C and 4°C, we collected confocal Raman spectra in the x-y plane and then scanned along z-direction (height of the cell) from -20 to 20 μm with a 1 μm z-step size. We arbitrarily designated z = 0 to be ~2 μm from the basal plane. We independently determined the cell height to be ~7 μm (z = -2 μm to z = 5 μm) using the laser scanning confocal microscope. SWCNTs for the 37°C sample were located in a z region approximately corresponding to the height of the cell (Figure 5C). Conversely, the 4°C sample did not show SWCNT intensity for height regions corresponding to the cell height (Figure 5C). Minimal SWCNT intensity was observed below z = -2 μm, corresponding to the SWCNTs on the substrate, and above z = 5 μm, corresponding to SWCNTs on the cell membrane.

### Altered endocytosis rates in SWCNT treated cells

**Figure 6:**
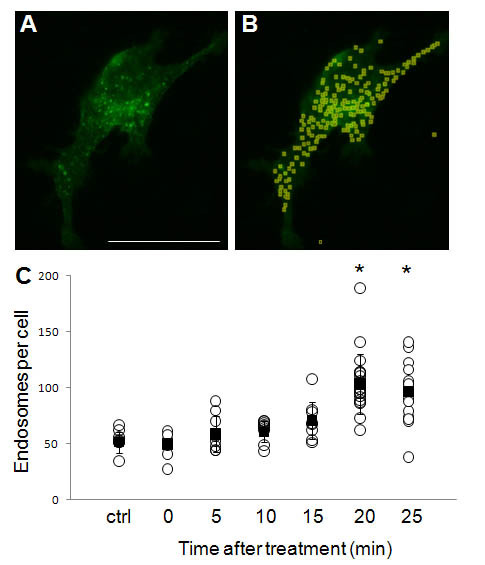
**Treatment with SWCNTs increases the endosomes per cell**. **(A) **Cells were transfected with GFP-endo (Rho-GTPase), a marker for endocytosis, and treated with 100 μg/mL SWCNTs. Cells were then fixed at set timepoints after treatment and rapidly imaged by widefield microscopy. **(B) **The observable endosomes were segmented and counted using ImageJ. Errant points, such as those outside the cell, were identified and removed from the counts. The number of endosomes was determined by an average of n = 30 cells per treatment over 2 treatments at all time points. **(C) **After treatment with SWCNTs there was an apparent increase in endosomes per cell. After 20 min, this change became statistically significant from control (p < 0.001). Open circles are individual cells and black boxes are the average with an SEM error bar.

We showed that the uptake of PF-127 stabilized SWCNTs was energy-dependent, but we also performed more direct measurements of endocytosis. Endocytosis in HeLa cells was visualized using a GFP-tagged RhoB-GTPase (pAcGFP-1-endo, Clontech, further referred to as GFP-endo), which labels endosomes in mammalian cells. Cells fixed at set time points after SWCNT treatment were imaged for endosomes (Figure 6A) and quantified using ImageJ (Figure 6B). While uncertainties in this measurement exist from exogenous expression of the marker, widefield imaging, and image analysis capturing potentially errant signal (see Methods), the control sample showed remarkable precision from cell to cell and experiment to experiment (Figure 6A, control and 0 min). We saw slight increases in endosome numbers per cell with increased time of exposure to SWCNTs, with a statistically significant increase in endosome number at 20 and 25 minutes. Thus, treatment with SWCNTs appeared to alter cellular endocytosis after 20 minutes.

### FLIM of endosomes

**Figure 7:**
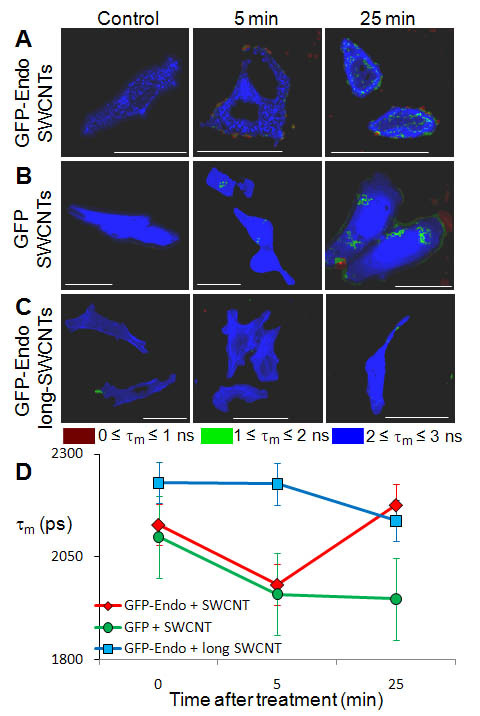
**FLIM of cytoplasmic GFP and GFP-tagged endosomes in cells exposed to SWCNTs**. **(A) **Addition of SWCNTs was associated with a decrease in GFP mean fluorescence lifetime (τ_m_, pseudo-colored red to green to blue). This change in τ_m _was most obvious at the periphery of the cell and moved inward over longer times, as seen by more internal green regions and a more severe quenching (green to red) at the periphery. **(B) **Expression of soluble GFP in the cytoplasm also showed a reduction in τ_m _upon incubation with SWCNTs. **(C) **Treatment of cells with long SWCNTs (1250 nm vs. 145 nm) showed little change in the GFP-endo τ_m_. **(D) **Quantification of the τ_m _revealed time-dependent behavior. The τ_m _of GFP-endo is significantly (p < 0.01) reduced after 5 min of SWCNT exposure but recovered to near control levels after 25 min of exposure. Similarly, the τ_m _of cytoplasmic GFP significantly (p < 0.01) decreased after 5 min of SWCNT treatment; however, unlike GFP-endo, it remained quenched after 25 min of exposure to SWCNTs. The τ_m _of GFP-endo, when exposed to long SWCNTs was relatively unchanged.

Using FLIM, we directly visualized SWCNT interaction with fluorescent endosomes. For all treatment conditions, the fluorescence lifetime of GFP and GFP-endo was best modeled as a double exponential decay (Tables 2 and 3). GFP and GFP-endo showed a majority τ_m _of 2-3 ns (Figure 7), which is consistent with previously reported FLIM of GFP in HeLa cells[37]. We treated cells expressing GFP (soluble in the cytoplasm) or GFP-endo with SWCNTs added at a final concentration of 100 μg/mL at t = 0 and followed the mean fluorescence lifetime, τ_m_, at 5 minutes and 25 minutes - before and after the observed changes in endosomes per cell shown in Figure 6. We saw a decrease in the τ_m _of GFP-endo after 5 minutes of treatment with SWCNTs (Figure 7A, 5 minutes, green spots at the periphery) and more significantly after 25 minutes (Figure 7A, 25 minutes, red and green spots). FLIM of GFP (in the cytoplasm) averaged over the cell showed a similar decrease in fluorescence lifetime at short time (Figure 7B, 5 min). To determine whether SWCNT length affected cellular uptake, we treated HeLa cells with long SWCNTs (1250 nm versus 145 nm) and found that the τ_m _for GFP-endo fluorescence was unchanged after the addition of long SWCNTs (Figure 7C).

**Table 3:** Fluorescence lifetime imaging microscopy (FLIM) values obtained from cells transfected with GFP-endo and GFP and exposed to SWCNTs.

GFP-endo (+) "Short" (145 ± 17 nm) SWCNTs						
	τ_1_	A_1_	τ_2_	A_2_	τ_m_	χ^2^
**Control**	1732 ± 65	0.574 ± 0.081	2784 ± 76	0.426 ± 0.074	2128 ± 37	1.200 ± 0.037
**5 min**.	1588 ± 117	0.601 ± 0.105	2757 ± 116	0.399 ± 0.068	1982 ± 56	1.291 ± 0.088
**25 min**.	1918 ± 124	0.553 ± 0.109	2560 ± 101	0.447 ± 0.104	2176 ± 58	1.346 ± 0.072

**GFP (+) "Short" (145 ± 17 nm) SWCNTs**						

	**τ_1_**	**A_1_**	**τ_2_**	**A_2_**	**τ_m_**	**χ^2^**
**Control**	1638 ± 83	0.558 ± 0.133	2803 ± 107	0.442 ± 0.125	2098 ± 75	1.261 ± 0.082
**5 min**.	1306 ± 146	0.624 ± 0.173	3054 ± 136	0.376 ± 0.132	1959 ± 98	1.364 ± 0.206
**25 min**.	1574 ± 186	0.568 ± 0.163	2527 ± 118	0.432 ± 0.138	1947 ± 86	1.342 ± 0.099

**GFP-endo (+) "Long" (1.25 ± 0.75 μm) SWCNTs**						

	**τ_1_**	**A_1_**	**τ_2_**	**A_2_**	**τ_m_**	**χ^2^**
**Control**	1945 ± 131	0.550 ± 0.111	2606 ± 107	0.450 ± 0.109	2230 ± 60	1.231 ± 0.063
**5 min**.	2026 ± 167	0.534 ± 0.110	2515 ± 121	0.466 ± 0.101	2227 ± 70	1.456 ± 0.101
**25 min**.	1827 ± 151	0.583 ± 0.097	2639 ± 111	0.417 ± 0.088	2137 ± 59	1.337 ± 0.064

Quantification of τ_m _from the different experimental conditions (Figure 7A-C) showed time-dependent changes (Figure 7D). Treatment with short SWCNTs generated a statistically significant (p < 0.01) reduction in τ_m _after 5 minutes for both GFP-endo and GFP compared to their respective controls. At 25 minutes after treatment, the GFP τ_m _remained significantly reduced. However, the GFP-endo τ_m _increased at 25 minutes. This suggests that HeLa cells had begun increasing the number of endosomes after 25 minutes (such as in Figure 6) which returned some of the signal to a more-baseline level. Also SWCNTs may have escaped from the endosomes, so they would no longer quench GFP-endo, but rather soluble GFP in the cytoplasm. The long SWCNTs showed no change in τ_m _at 5 minutes of treatment and only a slightly decrease in τ_m _at 25 minutes suggesting incubation with long SWCNTs does not alter the GFP fluorescence lifetime. Raw FLIM data is available in Table 3: A_1 _and A_2 _are the average relative contributions of τ_1 _and τ_2 _to τ_m _(Eq. 1 in Methods); average τ_m _cannot be calculated directly from averaging A_i _and τ_i_.

## Discussion

Previous studies involving several different cell types have demonstrated uptake of various types of functionalized CNTs[38-43]. Most studies show that CNTs enter cells via endocytosis[10-14]. However, given that millions of CNTs can enter cells[44], a small fraction of CNTs piercing the membrane could be significant. Some theoretical analyses suggested that SWCNTs may be insufficient to trigger endocytosis due to their small diameter and the kinetics of endosome formation[15,16]. Experiments using nanoparticles of varying size and composition determined that uptake of nanoparticles depends strongly on their size[11,15,45]. However, given the unique anisotropy of SWCNTs, it is unclear which orientation governs interactions with cells[16,46]. Using complementary membrane and cellular techniques we have shown unambiguously that polymer-dispersed SWCNTs were unable to penetrate complete bilayers. However, the affinity of the SWCNTs for membranes did appear to increase rates of endocytosis in the cell.

### Endocytosis of SWCNTs

Confocal Raman spectroscopy and imaging of HeLa cells revealed that SWCNTs were preferentially localized within the cell. SWCNTs were observed throughout the cell with SWCNT intensity located in the perinuclear region, consistent with previous observations[10,24], possibly suggesting that SWCNTs entered cells via endocytosis and were deposited in or near the endoplasmic reticulum. This is also supported by a reduction in cellular uptake of SWCNTs at 4°C when energy-dependent processes, including endocytosis, are reduced. We suggest that SWCNTs or PF-127 by means of their membrane activity were able to disrupt some of the endosomes as they shrank into lysosomes, thus depositing SWCNTs throughout the cell and enabling them to interact with other cellular components[9].

SWCNTs enter cells via an energy-dependent endocytosic process. However, endocytosis may proceed by a number of different mechanisms including clathrin-mediated endocytosis, calveoli-mediated endocytosis and pinocytosis (see review of nanomaterial uptake by endocytosis [45]). In this study we have not examined the specific type of endocytosis relevant for SWCNT uptake which could be further clarified by targeting integrins and other cell-specific receptors on the plasma membrane.

### SWCNTs associate with but do not penetrate membranes

Synthetic membrane experiments indicated that SWCNTs cannot completely penetrate membrane bilayers, but changes in monolayer pressure suggested that SWCNTs might penetrate the outer leaflet of lipids. We hypothesize that the association of SWCNT with lipids may alter membrane tension locally and stimulate endocytosis[47,48]. Exposure of cells to SWCNTs significantly increased the number of endosomes when compared to control (Figure 4). Therefore, we suggest that SWCNTs affect the tension of the cell membrane, which may lead to a significant increase in endocytotic activity.

We have shown that short (145 nm) SWCNTs altered the fluorescence lifetime of GFP-endo, which label endosomes. While it is possible that these SWCNTs only indirectly alter the fluorophore's nanoenvironment, we have also shown that long (1,250 nm) SWCNTs had no effect on lifetime compared to control, and these long SWCNTs are too long for endocytosis[11,15,45]. Further, confocal Raman imaging confirmed that short SWCNTs entered HeLa cells, probably via endocytosis as evidenced by the perinuclear localization. We propose that short SWCNTs in endosomes directly altered the τ_m _of the GFP-endo fluorophore; long SWCNTs, unable to enter via endocytosis, did not alter the τ_m _of GFP-endo. Soluble GFP was quenched 5 and 25 min after treatment, suggesting that SWCNTs were liberated from endosomes and quenched non-localized cellular GFP, further confirming the SWCNT sub-cellular localization, independently demonstrated via confocal Raman imaging. Unlike the GFP-endo, the GFP remained quenched after 25 min, suggesting that some endosomes may have ruptured and released SWCNTs into the intracellular space and that the rate of entry of SWCNTs into the cell is decreased at long time.

### Potential mechanisms of SWCNT uptake

In combination, the wide range of techniques used in this work shows conclusively that short Pluronic-coated SWCNTs enter the cell via endocytosis and not via membrane penetration. From our results we suggest that the mechanism of SWCNT uptake into cells includes the following steps: (A) SWCNTs adsorb onto the cell surface and penetrate into the outer leaflet of the bilayer. (B) This association increases membrane tension and induces an imbalance between the outer leaflet and the inner leaflet. (C) Localized disturbances in membrane tension can stimulate endocytosis as the membrane attempts to regulate tension. (D) Once endocytosis is stimulated, the SWCNTs localize in the endosomes, which shrink during processing. (E) By disruption of the endosomes or lack of lysosomal processing, SWCNTs enter the cytoplasm.

The fate of the Pluronic polymer outside or inside the cell remains unknown. Polymer-coated SWCNTs have been shown to adsorb protein when injected into the circulation of animals[49]. We did not observe the Pluronic being displaced by protein in solution over 6 days with agitation (Additional Figure 2). However, the complex mixture of different proteins, surfactants and cellular secretions in the circulation and in cell culture media may prove to displace Pluronic from the SWCNTs. As such we cannot assess at what point PF-127 is lost from the tubes.

The pluronic coating likely increases the association of the SWCNTs with membranes, enhancing endocytosis. Since surfactant molecules are generally used to disperse SWCNTs in solutions, we believe that our results are generic features of SWCNT uptake into cells. The specific targeting of SWCNTs for use in therapeutics should also focus on endocytosis as the preferential method for the cellular uptake.

## Conclusions

We have shown that short, polymer-dispersed SWCNTs enter the cells by endocytosis, and SWCNTs do not penetrate through bilayers non-specifically. There is a preferential interaction of SWCNTs with membranes, which likely increases rates of endocytosis after ~30 minutes. Thus the membrane affinity of SWCNTs control cellular uptake, but incorporation does not occur by passive membrane penetration.

## Methods

### Synthesis of SWCNTs

Details of the synthesis and dispersion of SWCNTs[9,19] are described in detail in the Additional Methods.

### Cell Culture and Imaging

HeLa cells were grown in Dulbecco's Modified Eagle Medium (DMEM; Hyclone catalog number SH30021) with 10% fetal bovine serum (FBS) and 1% penicillin/streptomycin (both Invitrogen), collectively referred to as cell culture media (CCM). Cells were passaged onto #1.5 coverslips in 35 mm dishes, incubated for 12 - 24 hours and exposed to SWCNTs in 2 mL of CCM. Solutions were equally distributed over the cells, and CCM was pre-heated for most experiments. For 4°C cell culture experiments, the CCM was removed and the cells washed with phosphate buffer saline (PBS) at room temperature before 2 mL of 4°C CCM with SWCNTs was added, and the plate was immediately transferred to a 4°C cold-room for 15 min.

Cells were transferred to coverslips in 35 mm dishes or 6-well plates and were treated with 1 mL of SWCNTs diluted in CCM; SWCNT concentrations and times are reported in the results section for particular experiments. For imaging, cells on coverslips were washed twice with PBS at room temperature, fixed for 10 min with 3.7% formaldehyde (Sigma-Aldrich) and mounted onto slides. For some Raman imaging experiments, hematoxylin (Sigma-Aldrich) was added before mounting to enhance contrast of cellular features, but most cells were not labeled.

Brightfield and widefield fluorescence imaging of the cells was performed with 40 × (oil, 1.25 N.A.) or 63 × (oil, 1.4 N.A.) objectives on a Leica DMI6000 B inverted microscope. Confocal Raman spectroscopy was performed on an inverted Raman confocal microscope (inVia, Renishaw) with a 785 nm laser (100 mW) using either a 40 × (oil, 1.25 N.A.) or 100 × (oil, 1.4 N.A.) objective (Leica Microsystems). The in-plane (x-y) resolution of the Raman microscope with the 100 × objective is ~250 nm and the z resolution is ~300 nm. Confocal Raman spectra between 1327 and 1819 cm^-1 ^were collected with a 0.82 cm^-1 ^resolution. We then quantified the Raman intensity at 1590 ± 17 cm^-1 ^to obtain intensity maps of SWCNTs; the tangential mode of graphene, called the G-band, is located at 1590 cm^-1 ^and is widely used to identify SWCNTs[13,23]. Image analysis was performed using WiRE (Renishaw), and z-section data were analyzed using MATLAB (MathWorks) code developed in the lab.

### Cell Transfection and Imaging of Endosomes

For transient transfection, cells were grown to ~ 60% confluence and transfected with either pAc-GFP1-Endo or pEGFP-C1 (Clontech) using PolyFect (Qiagen) according to manufacturer's recommendations, and cells were incubated overnight. To measure changes in endocytosis, cells were incubated with 100 μg of SWCNTs in CCM for 0 (added and immediately washed), 5, 10, 15, 20 and 25 min at 37°C in the tissue culture incubator. At the selected time points, the CCM with SWCNTs was removed, and cells were washed, fixed and mounted as described above. All procedures were performed in low light to prevent GFP photobleaching during incubation, fixation, mounting, and transportation.

Confocal imaging caused photobleaching of small spots and could not be used to image endosomes in the entire cell, so we used widefield fluorescence imaging. To minimize artifacts of out-of-plane light, cells were imaged in the central plane, as identified by the z-position at which the nucleus was in best focus. Previous analysis has shown that there was no change in overall height in SWCNT-treated cells[9]. Images were processed with ImageJ to identify GFP-labeled endosomes by their fluorescence intensity and minimum and maximum sizes.

### Giant Plasma Membrane Vesicles (GPMVs)

Vesicles were produced from NIH-3T3 cells grown in DMEM (Hyclone SH30022) supplemented with 10% calf serum and 1% penicillin/streptomycin in 60 mm dishes at 80-90% confluence using a method described elsewhere[50,51]. Briefly, each dish was washed twice with 10 mL of a freshly prepared and thoroughly mixed buffer containing 10 mM HEPES, 0.15 M NaCl, 2 mM CaCl_2_, and 0.05% v/v gelatin. Cells were then incubated in 5 mL of buffer plus 1 mM dithiotheritol (DTT) and 25 mM formaldehyde for 1 hour at 37°C on an orbital shaker at 75 rpm. The solution was gently decanted and allowed to sit for 15 min at 4°C in a conical tube. Samples were taken from the middle portion of the solution to minimize extracellular material and necrotic cells. GPMVs were imaged directly using widefield microscopy or were treated with the membrane-active CellMask orange fluorophore (Invitrogen) with gentle agitation for 5 min. Then, 0.5 mL of the GPMV solution was transferred to a 35 mm glass-bottom dish (MatTek) pre-incubated with poly-L-lysine solution (Sigma-Aldrich).

### Fluorescence Lifetime Imaging (FLIM)

FLIM was performed using a Leica TCS SP5 inverted laser scanning confocal microscope with a 100 × (oil, 1.4 N.A.) objective with a pixel resolution of 256 × 256 and a scan rate of 400 Hz. A tunable (720-950 nm), mode-locked Ti:sapphire pulsed infrared laser (Chameleon, Coherent) served as the multiphoton (MP) excitation source (1 W, average). Pulse-widths of < 140 fs were delivered at 90 MHz. For GFP, the MP laser was tuned to 942 nm, and the FLIM-dedicated photomultiplier tube (PMT) was tuned to 481-615 nm to detect the full range of GFP emission. For CellMask orange, the MP laser was tuned to 900 nm with the PMT tuned to 550-700 nm.

Time-correlated single photon counting (TCSPC) was implemented using a Becker & Hickl SPC-830 acquisition package with 10 ps resolution that includes three software programs: DCC (controls DCC 100 hardware), SPCM (controls image acquisition), and SPCImage (controls lifetime data analysis). Lifetime images were acquired for 180 s to minimize the coefficient of variation to ~2.5%[52,53]. 220 time channels and a measurement window of 10.8 ns were used to minimize the variance of lifetime over a wide range of ratios of the measurement window to lifetime[53]. The rates of the detected, converted, and stored photons were maintained between 1 × 10^4 ^and 1 × 10^6 ^(< 1% of laser repetition rate) to prevent errors in lifetime determination due to the pileup effect[53], to achieve the required signal-to-noise ratio after 3 min of detection, and to minimize photobleaching.

### FLIM Data Processing

Fluorescence lifetime analysis was performed using SPCImage (Becker & Hickl). Instrument response functions were estimated and manually verified through the SPCImage software for each data set[53]. Lifetime images were binned to achieve a peak photon count of ≥ 1000 (≥ 50,000 photons over all time channels), ensuring that two exponential decays could be accurately resolved[53,54].

Fluorescence lifetime is modeled as a sum of exponential decays, taking the form:

(Eq.1) I(t)=I0+ ∑nan exp-tτn

where *I(t) *is the number of photons detected per time, I_0 _is a background offset, a_n _is the normalized amplitude, and τ_n _is the lifetime of the n^th ^exponential decay. For multi-exponential decays, a mean lifetime, τ_m_, is defined as

(Eq.2) $$\tau_{m}=\frac{\underset{n}{\sum }a_{n}\tau_{n}}{\underset{n}{\sum }a_{n}}$$

Each pixel was separately modeled as a single and a double exponential decay, and the corresponding average lifetimes (τ_1_, τ_2 _and τ_m_), goodness of fits (χ^2^), and standard deviations were calculated in MATLAB. Reported lifetime values (Tables 2 and 3) were averaged and errors were calculated using the derivative method of error propagation. Thus, the spatially average values presented in Table 3 will comply with Eq. 2 for the individual values. Fluorescence lifetime images were then generated using SPCImage.

### Sparsely-Tethered Bilayer Lipid Membranes (stBLMs)

The synthetic, zwitterionic phospholipids used in the model membrane experiments, i.e., dipalmitoyl-sn-glycro-3-phosphocholine and dioleoyl-sn-glycero-3-phosphocholine (DPPC and DOPC, respectively), were from Avanti Polar Lipids. stBLMs were prepared on gold-coated Si wafers by precipitation of DOPC onto a preformed self-assembled monolayer (SAM) as described[25]. For further details, see Additional Methods.

### Electrochemical Impedance Spectroscopy (EIS)

EI spectra of stBLMs on Si/gold in Teflon (PTFE) sample cells of local design were obtained between 1 Hz and 65 kHz with ten logarithmically distributed measurements per decade using a Solartron (Farnborough) potentiostat and frequency analyzer. The gold-coated Si wafer at the bottom of a buffer-filled compartment served as the electrode in connection with a saturated [Ag|AgCl|NaCl] reference and a Pt auxiliary electrode immersed into the buffer. Details are given in Additional Methods.

Fitting of the EI spectra was performed using the electrical equivalent circuit model (ECM) represented in Additional Figure 1B. CPE, or constant-phase element, refers to an electrical element with an impedance, Z_CPE _= 1/T(iω)^α^, where T is a coefficient measured in Farad per unit area × s^α-1^, and the exponent α varies between 0 and 1[25]. The ECM in Additional Figure 1B shows two CPEs, one associated with the capacitive nature of the bilayer (CPE_stBLM_) and one associated with the capacitive properties of defects in the bilayer (CPE_defect_). Such defects also give rise to a residual conductance, Y = 1/*R*_defect_. In addition, *R*_solvent _and *C*_stray _describe the resistivity of the buffer and stray capacitance of the sample configuration. Two ECM parameters, CPE_stBLM _and *R*_defect_, obtained from the model were used to quantify the quality of as-prepared bilayers and changes in bilayer properties upon incubation with PF-127-dispersed SWCNTs or, as a control, equivalent concentrations of PF-127 alone. If the CPE exponent α equals 1, the CPE simply reduces to the capacitance, *C*. Since this is the case here (see Table 3), the two ECM parameters represent the bilayer capacitance and the inverse of the residual conductance due to defects.

### Langmuir Monolayers (LMs)

A custom-built Teflon trough (Nima Technology, U.K.) with a surface area of 250 cm^2 ^and a depth of 0.5 cm on an active anti-vibration table was filled with filtered H_2_O (Milli-Q, Millipore). The surface pressure, Π, due to a deposited LM was monitored using a Wilhelmy plate, cut from ashfree filter paper and connected to an electronic microbalance. LMs were formed by spreading either DPPC or DOPC dissolved in chloroform using a microsyringe (Hamilton) and allowing the solvent to evaporate for at least 15 min. The resulting LMs were compressed by two symmetrically moving barriers to a desired surface pressure, Π_0_. Subsequently, dispersed SWCNT in aqueous solution were injected into the subphase underneath a LM and the ensuing surface pressure change ΔΠ(t) was recorded at constant surface area over time, t, until it reached at stable plateau. Such experiments, performed at a series of increasing values of Π_0_, were used to determine the maximum insertion pressure (MIP), i.e., the minimum value of Π_0 _that just suppresses any insertion of the adsorbent into the LM.

## Abbreviations

CPE: constant-phase element; DMEM: Dulbecco's Modified Eagle Medium; DOPC: 1,2-dioleoyl-sn-glycero-3-phosphocholine; DPPC: 1,2-dipalmitoyl-*sn*-glycero-3-phosphocholine; ECM: equivalent circuit model; EIS: electrical impedance spectroscopy; FBS: fetal bovine serum; FLIM: fluorescence lifetime imaging microscopy; GPMV: giant plasma membrane vesicle; LM: Langmuir monolayer; PBS: phosphate buffer saline; stBLM: sparsely tethered bilayer lipid membrane; SWCNT: single wall carbon nanotube.

## Competing interests

The authors declare that they have no competing interests.

## Authors' contributions

PNY performed membrane and GPMV experiments and prepared manuscript. BDH performed FLIM and Raman experiments. PAS performed endocytosis experiments and transfected cells for FLIM experiments. ML helped design and analyze membrane experiments. MFI provided SWCNTS and helped design and analyze FLIM and Raman experiments. KND provided cells, reagents and rDNA and helped design and analyze cellular experiments. All authors have read and approved the final manuscript.

## Supplementary Material

Additional File 1 **Additional Methods: SWCNT preparation, Sparsely tethered bilayer lipid membranes (stBLM) preparation, Electrochemical impedance spectroscopy (EIS)**. Additional Table 1: Model-fits of data using the equivalent circuit model (ECM) shown in Additional Figure 1B for DOPC stBLMs. Additional Figure 1: Schematic of the stBLM configuration and model used for fitting. Additional Figure 2: Comparison of vis-NIR spectra suggests that proteins do not displace PF-127. Click here for file
