# Supplementary material for: Single wall carbon nanotubes enter cells by endocytosis and not membrane penetration
Source: J Nanobiotechnology. 2011 Sep 30;9:45. doi: 10.1186/1477-3155-9-45 (PMC3195092; doi:10.1186/1477-3155-9-45)
Supplement: Additional File 1 — Additional Methods: SWCNT preparation, Sparsely tethered bilayer lipid membranes (stBLM) preparation, Electrochemical impedance spectroscopy (EIS). Additional Table 1: Model-fits of data using the equivalent circuit model (ECM) shown in Additional Figure 1B for DOPC stBLMs. Additional Figure 1: Schematic of the stBLM configuration and model used for fitting. Additional Figure 2: Comparison of vis-NIR spectra suggests that proteins do not displace PF-127. [file 1477-3155-9-45-S1.DOC]

Additional Methods

SWCNT preparation

We used two species of SWCNTs termed “short” and “long” for clarity and convenience. The short SWCNTs were produced via HiPCO (high-pressure carbon monoxide conversion synthesis) and long SWCNTs by CoMoCAT (Cobalt / Molybdenum catalysis) methods. We have previously reported our protocols for SWCNT purification, length selection, and dispersion[9]. Briefly, we purified the SWCNTs such that the SWCNT sample contained <5 wt% carbonaceous impurities, ~0.3% metallic impurities, and the rest SWCNTs[18]. Length-fractionation was achieved using a density gradient of DOC-dispersed[19] SWCNTs in water. Note all water used in our experiments was Millipore filtered deionized water of resistivity 18.3 MΩ·cm^-1^. After fractionation, the DOC was burned off, and then the SWCNTs were dispersed in water using Pluronic F127 (PF-127) (BASF), a biocompatible triblock copolymer (polyethylene oxide (PEO) – polypropylene oxide (PPO) – PEO). Centrifugation removed bundles from the PF-127-dispersed SWCNT solution, and the concentration of SWCNTs in the supernatant was determined using near-infrared (NIR) absorbance spectroscopy and an extinction coefficient of 2.6 (absorbance mL)/(mg mm) at 930 nm[18]. Sharp van Hove peaks in the absorption spectra indicated that SWCNTs were isolated in the dispersed supernatant[55].

Sparsely tethered bilayer lipid membranes (stBLM)

Gold layers of typically 100 Å thickness were deposited by high-energy magnetron (BOC Edwards) sputtering onto glass slides (Fisher Scientific). They had an RMS surface roughness of ~5 Å, as measured by x-ray reflectometry (Bruker AXS), and a uniformity of thickness across the surface of ±3% or better, as determined using ellipsometry. A self-assembled monolayer (SAM) was produced on a freshly prepared gold surface from an ethanolic solution of a tethered lipid[56] and βME (distilled β-mercaptoethanol from Sigma-Aldrich) at a 30:70 ratio and total concentration of 0.2 mM. The tether lipid, HC18, is similar to the FC16 compound recently described in detail, but incorporates two monounsaturated oleyl chains. It was synthesized by Dr. D. Vanderah at the National Institute of Standards and Technology (Chemical Sciences and Technology Laboratory, CSTL). After 12-36 hours of incubation, surfaces were rinsed with absolute ethanol and dried in a N_2_ gas stream. Bilayer formation by rapid solvent exchange[22] completed the synthetic stBLM: 150 μl of a 10 mM solution of DOPC (1,2-dioleoyl-sn-glycero-3-phosphocholine; Avanti Polar Lipids) in 100% ethanol was used to incubate the SAM-covered substrate for 1 hour at room temperature. The physical characterization of the resulting stBLM is described elsewhere[56]. The lipid solution was then rapidly displaced by a large excess (> 30 ml) of buffer solution, taking care to avoid the formation of air bubbles at the surface. The buffer used was either 10 mM Tris-HCl (tris-hydroxymethyl aminomethane-HCl) or 10 mM phosphate buffer, pH 7.4–7.8, with NaCl (0.1 – 0.2 M). All preparation steps were performed at room temperature (21 ± 1° C).

Electrochemical impedance spectroscopy (EIS)

EIS data were taken using a Solartron system (model 1287A) electrochemical interface potentiostat and model 1252A frequency response analyzer and fitted to equivalent circuit models (ECMs; Additional Figure 1B) using ZView (Scribner Associates, Southern Associates, NC). Au-coated glass slides served as the work electrode and substrate for the stBLMs[57] in a setup with 6 different cells (volume: 250 – 300 μl). For quality control and cross-sample comparison purposes, 1 cell was left unexposed to measure the quality of the SAM and 1 cell was left with an undisturbed bilayer. Each cell had a surface area of 0.33 cm^2^ that was confined by a Viton O-ring used to make a liquid-tight seal with the electrode surface. EIS data were normalized to the cell surface area and the capacitance and resistance of the leads have been subtracted out. A saturated silver-silver chloride electrode was used (Microelectrodes, model M-401-F) as the reference. The auxiliary electrode was a 0.25-mm-diameter Pt wire (99.999% purity, Aldrich) coiled around the barrel of the reference electrode. The distance between the reference and Au-electrode surface was set to ~2.5 mm. Measurements were carried out at 10 mV a.c. with 0 V bias versus the reference electrode.

Spectra of the stBLMs before and after exposure to SWCNTs were modeled using a procedure based on the ECM (Additional Table 1 and 2). The model contains 5 independent elements where each CPE in the circuit is represented by two values, representing the CPE coefficient T and the exponent α of Z_CPE_. Confidence limits of the best-fit model parameters were quantified by evaluating the variance-covariance matrices of the Levenberg-Marquardt algorithm employed in the nonlinear-χ^2^ minimization.

Additional Table 1: Model-fits of data using the equivalent circuit model (ECM) shown in Additional Figure S1B for DOPC stBLMs

| Volume SWCNTs treatment time | C_stray_  (μFcm^-2^s^α−1^) | C_stray_  (Error) | R_series_  (kΩcm^2^) | R_series_  (Error) | CPE_stBLM_-T  (μFcm^-2^s^a-1^) | CPE_stBLM_-T  (Error) | α_stBLM_  | α  (Error)  |
| --- | --- | --- | --- | --- | --- | --- | --- | --- |
| stBLM control | 1.28E-02 | 2.24E-04 | 1.03E-01 | 1.81E-04 | 1.11 | 5.52E-03 | 0.984 | 7.03E-04 |
| 25 ul 0 min | 2.03E-02 | 3.35E-04 | 8.06E-02 | 1.72E-04 | 1.12 | 6.12E-03 | 0.982 | 7.58E-04 |
| 25 ul 30 min | 2.72E-02 | 4.11E-04 | 7.29E-02 | 1.76E-04 | 1.13 | 6.62E-03 | 0.982 | 8.14E-04 |
| 25 ul 18 hrs | 2.46E-02 | 3.89E-04 | 6.50E-02 | 1.34E-04 | 1.13 | 5.70E-03 | 0.983 | 6.89E-04 |
| stBLM control | 1.35E-02 | 2.40E-04 | 9.93E-02 | 1.81E-04 | 1.11 | 5.43E-03 | 0.984 | 6.95E-04 |
| 50 ul 0 min | 1.82E-02 | 2.85E-04 | 8.81E-02 | 1.72E-04 | 1.13 | 5.61E-03 | 0.983 | 7.04E-04 |
| 50 ul 30 min | 2.46E-02 | 3.10E-04 | 8.09E-02 | 1.59E-04 | 1.12 | 5.44E-03 | 0.983 | 6.81E-04 |
| 50 ul 18 hrs | 2.42E-02 | 3.35E-04 | 7.05E-02 | 1.34E-04 | 1.13 | 5.11E-03 | 0.982 | 6.28E-04 |
| stBLM control | 1.40E-02 | 3.99E-04 | 1.06E-01 | 3.45E-04 | 1.10 | 8.80E-03 | 0.978 | 1.16E-03 |
| 25 ul 0 min | 1.92E-02 | 4.66E-04 | 9.67E-02 | 3.39E-04 | 1.11 | 9.16E-03 | 0.979 | 1.19E-03 |
| 100 ul 30 min | 2.27E-02 | 3.80E-04 | 8.83E-02 | 2.32E-04 | 1.12 | 6.85E-03 | 0.981 | 8.97E-04 |
| 100 ul 18 hrs | 2.25E-02 | 4.46E-04 | 8.10E-02 | 2.34E-04 | 1.11 | 7.71E-03 | 0.981 | 9.92E-04 |
| average stBLM control | **1.34E-02** | **5.17E-04** | **1.03E-01** | **4.29E-04** | **1.10E+00** | **1.17E-02** | **0.982** | **1.53E-03** |

Additional Table 1 (continued)

| Volume SWCNTs treatment time | χ^2^  | R_defect_ (kΩcm^2^) | R_defect_  (Error) | CPE_defect_-T (μFcm^-2^s^α−1^) | CPE_defect_-T  (Error) | α_defect_  | α  (Error)  |
| --- | --- | --- | --- | --- | --- | --- | --- |
| stBLM control | 3.16E-05 | 34.7 | 0.358 | 11.8 | 0.239 | 0.714 | 1.22E-02 |
| 25 ul 0 min. | 4.13E-05 | 36.6 | 0.425 | 11.6 | 0.285 | 0.725 | 1.47E-02 |
| 25 ul 30 min. | 4.84E-05 | 40.7 | 0.595 | 11.4 | 0.320 | 0.692 | 1.79E-02 |
| 25 ul 18 hrs. | 3.44E-05 | 44.1 | 0.702 | 11.2 | 0.262 | 0.633 | 1.69E-02 |
| stBLM control | 3.38E-05 | 38.2 | 0.377 | 12.1 | 0.299 | 0.748 | 1.45E-02 |
| 50 ul 0 min. | 3.64E-05 | 40.4 | 0.431 | 11.9 | 0.317 | 0.743 | 1.58E-02 |
| 50 ul 30 min. | 3.46E-05 | 45.3 | 0.553 | 11.8 | 0.329 | 0.706 | 1.78E-02 |
| 50 ul 18 hrs. | 3.06E-05 | 51.2 | 0.709 | 11.9 | 0.334 | 0.665 | 1.98E-02 |
| stBLM control | 1.07E-04 | 67.4 | 1.53 | 11.0 | 0.867 | 0.753 | 4.99E-02 |
| 25 ul 0 min. | 1.17E-04 | 73.4 | 1.82 | 10.7 | 0.977 | 0.758 | 5.80E-02 |
| 100 ul 30 min. | 6.29E-05 | 73.5 | 1.73 | 11.4 | 0.662 | 0.661 | 4.39E-02 |
| 100 ul 18 hrs. | 7.32E-05 | 84.4 | 3.20 | 10.5 | 0.602 | 0.576 | 5.53E-02 |
| average stBLM controls | **5.76E-05** | **46.8** | **1.62** | **11.6** | **0.948** | **0.738** | **5.34E-02** |


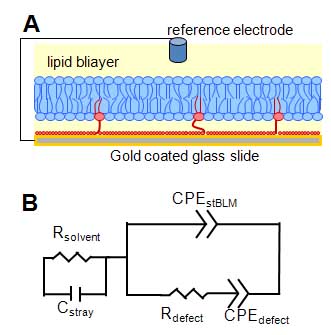


Additional Figure S1: (A) A schematic of the stBLM configuration in the EIS measurements. (B) Equivalent circuit model (ECM) used for fitting the EIS spectra.

Additional **Figure S2: Comparison of vis-NIR spectra suggests that proteins do not displace PF-127.** PF-127 dispersed SWCNTs (gray line, before BSA) were measured by vis-NIR spectra. These SWCNTs were then added 1:1 with a 1 wt% solution of bovine serum albumin (BSA) for a final protein concentration of 5 mg/mL, which is the maximum protein concentration for the cell culture media used here. The vis-NIR spectra for SWCNTs incubated with BSA protein for increasing time (red, orange, green and blue lines) were diluted by half, but did not change in characteristic spectra. The solution was then sonicated for 45 minutes, but this agitation did not cause a change in the spectra (purple line). The vis-NIR spectra of SWNCTs dispersed into BSA directly is shown as an overlay (black line).
